# AMPA Receptor Antagonists Facilitate NEDD4-2-Mediated GRIA1 Ubiquitination by Regulating PP2B-ERK1/2-SGK1 Pathway in Chronic Epilepsy Rats

**DOI:** 10.3390/biomedicines9081069

**Published:** 2021-08-23

**Authors:** Ji-Eun Kim, Duk-Shin Lee, Hana Park, Tae-Hyun Kim, Tae-Cheon Kang

**Affiliations:** Department of Anatomy and Neurobiology, Institute of Epilepsy Research, College of Medicine, Hallym University, Chuncheon 24252, Korea; dslee84@hallym.ac.kr (D.-S.L.); M19050@hallym.ac.kr (H.P.); hyun1028@hallym.ac.kr (T.-H.K.)

**Keywords:** 3-phosphoinositide-dependent protein kinase-1, AKT, cyclosporin A, GluA1, GluR1, intractable epilepsy, PDK1, refractory seizure

## Abstract

The neural precursor cell expressed by developmentally downregulated gene 4-2 (NEDD4-2) is a ubiquitin E3 ligase that has a high affinity toward binding and ubiquitinating glutamate ionotropic receptor α-amino-3-hydroxy-5-methyl-4-isoxazolepropionic acid (AMPA) type subunit 1 (GRIA1, also referred to GluR1 or GluA1). Since dysregulation of GRIA1 surface expression is relevant to the responsiveness to AMPA receptor (AMPAR) antagonists (perampanel and GYKI 52466) in chronic epilepsy rats, it is likely that NEDD4-2 may be involved in the pathogenesis of intractable epilepsy. However, the role of NEDD4-2-mediated GRIA1 ubiquitination in refractory seizures to AMPAR antagonists is still unknown. In the present study, both AMPAR antagonists recovered the impaired GRIA1 ubiquitination by regulating protein phosphatase 2B (PP2B)-extracellular signal-regulated kinase 1/2 (ERK1/2)-serum and glucocorticoid-regulated kinase 1 (SGK1)-NEDD4-2 signaling pathway in responders (whose seizure activities are responsive to AMPAR), but not non-responders (whose seizure activities were uncontrolled by AMPAR antagonists). In addition, cyclosporin A (CsA, a PP2B inhibitor) co-treatment improved the effects of AMPAR antagonists in non-responders, independent of AKT signaling pathway. Therefore, our findings suggest that dysregulation of PP2B-ERK1/2-SGK1-NEDD4-2-mediated GRIA1 ubiquitination may be responsible for refractory seizures and that this pathway may be a potential therapeutic target for improving the treatment of intractable epilepsy in response to AMPAR antagonists.

## 1. Introduction

Epilepsy is a brain function disorder characterized by recurrent and unprovoked seizures. The prevalence of epilepsy in the general population is approximately 0.6−0.8% [1]. Initial/acute prolonged seizure (status epilepticus, SE), trauma, stroke or infections are postulated as precipitating factors of epilepsy [2]. Mesial temporal lobe epilepsy (MTLE) is the most common form of epilepsy and a medically intractable syndrome that is partially or totally uncontrolled by conventional anti-epileptic drug (AED) treatments [3]. Although disturbances in glutamatergic/GABAergic transmissions and the related signaling pathways are associated with the pathogenesis of MTLE in humans, the underlying mechanisms of MTLE remain largely unclear.

The pilocarpine model (including LiCI-pilocarpine model) serves as a reliable animal model of intractable epilepsy. The profiles of spontaneous recurrent seizures in this model resemble those of human MTLE. This model shows limbic seizures that become secondarily generalized, evolving to SE, which lasts for several hours (acute period). The SE is followed by a latent “seizure-free” period (about 15−30 days) and by a chronic period characterized by the presence of spontaneous recurrent seizures. The lesions of mesial temporal structures, including a well-known hippocampal sclerosis, in this model are also similar to those of human MTLE patients [4,5]. Therefore, the pilocarpine model provides the opportunity to investigate the pathogenesis of MTLE. 

The α-amino-3-hydroxy-5-methyl-4-isoxazolepropionic acid receptor (AMPAR) is one of the major subtypes of ionotropic glutamate receptors. AMPARs are comprised of combinations of glutamate ionotropic receptor AMPA type subunit 1 (GRIA1, also referred to GluR1 or GluA1)—GRIA4, which are assembled as homo- or heterotetramers [6]. The regulation of AMPAR trafficking is critical for homeostatic regulation of synaptic strength and the pathogenesis of epilepsy [7]. Indeed, AMPAR antagonists inhibit seizure activity in chronic epilepsy rats, accompanied by reduced GRIA1 surface expression in the hippocampus [4,8,9,10].

The neural precursor cell expressed by developmentally downregulated gene 4-2 (NEDD4-2) is a ubiquitin E3 ligase that has a high affinity toward binding and ubiquitinating membrane proteins [11]. Some neuronal membrane receptors/channels have been identified as substrates of NEDD4-2: voltage-gated Na^+^ channel (Na_v_)1.6 [12], voltage-gated K^+^ channels K_v_7/KCNQ [13,14,15] and neurotrophin receptor TrkA [16]. NEDD4-2 also regulates neuronal activity and seizure susceptibility through ubiquitination of the GRIA1 subunit of AMPAR [11,17]. Therefore, it is likely that NEDD4-2 may be involved in the fine-tuning of AMPAR-mediated neuronal excitation. Indeed, at least three missense mutations in the *NEDD4-2* gene are identified through genomic mutation screening in patients with epilepsy [18,19,20]. Furthermore, NEDD4-2 plays an important role in seizure progression in response to kainic acid through regulation of AMPAR ubiquitination [11,17,21,22]. 

On the other hand, multifactorial events are involved in the underlying mechanisms of pharmacoresistant epilepsy: (1) a reduced yield of AED concentration in the brain by hyper-activation of drug efflux transporter or sustained inflammatory conditions [23,24], (2) dysfunctions of ion/neurotransmitter channels of transporters [25], and (3) abnormal neural networks [25]. Interestingly, GRIA1 surface expression is higher in the hippocampus of chronic epilepsy rats than that of normal rats, which is attenuated by AMPAR antagonists (such as perampanel and GYKI 52466) in responders whose seizure activities are reduced by them [4,10,26]. Since ubiquitination of GRIA1 is linked to AMPAR surface expression and trafficking [27,28,29], it is postulated that AMPAR antagonists may modulate NEDD4-2 activity that is required for limiting GluA1 surface expression and functionality of AMPAR in the epileptic hippocampus. However, little data are available to describe whether NEDD4-2-mediated GRIA1 ubiquitination is changed, and this alteration is relevant to the generation of refractory seizures to AMPAR antagonists in a chronic epilepsy model. In the present study, therefore, we investigated the effects of AMPAR antagonists on NEDD4-2-mediated GRIA1 regulation in responders and non-responders (whose seizure activities were uncontrolled by AMPAR antagonists) of a LiCl-pilocarpine epilepsy rat model to elucidate the role of NEDD4-2 in MTLE. 

Here, we demonstrate that the anti-convulsive effects of AMPAR antagonists are closely related to the regulation of GRIA1 ubiquitination via protein phosphatase 2B (PP2B)-extracellular signal-regulated kinase 1/2 (ERK1/2)-serum and glucocorticoid-regulated kinase 1 (SGK1)-NEDD4-2 signaling pathway. In addition, impairment of this signaling pathway resulted in refractory seizures to AMPAR antagonists, which was improved by cyclosporin A (CsA, a PP2B inhibitor) co-treatment. Therefore, our findings suggest that the PP2B-ERK1/2-SGK1-NEDD4-2 pathway may be a potential therapeutic strategy to improve the treatment of intractable MTLE in response to AMPAR antagonists.

## 2. Materials and Methods

### 2.1. Experimental Animals and Chemicals

Male Sprague Dawley (SD) rats (seven weeks old) were provided with a commercial diet and water ad libitum under controlled temperature, humidity and lighting conditions (22 ± 2 °C, 55 ± 5% and a 12:12 light/dark cycle with lights). Animal protocols were approved by the Institutional Animal Care and Use Committee of Hallym University (Code number: #Hallym 2018-2, 26 April 2018, #Hallym 2018-21, 8 June 2018 and #Hallym 2021-3, 27 April 2021). All reagents were purchased from Sigma-Aldrich (St. Louis, MO, USA), except where noted.

### 2.2. Generation of Chronic Epilepsy Rats

**Figure 1:**
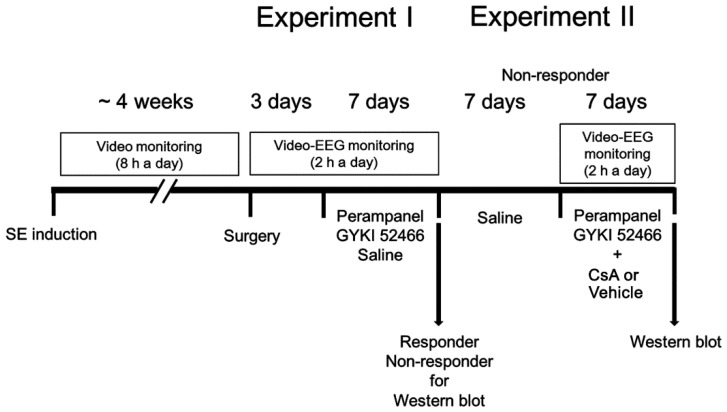
Scheme of the experimental design in the present study.

Animals were intraperitoneally (i.p.) given LiCl (127 mg/kg) 24 h before pilocarpine treatment. On the next day, animals were treated with pilocarpine (30 mg/kg, i.p.) 20 min after atropine methylbromide (5 mg/kg i.p.). Two hours after SE on-set, animals were administered diazepam (Valium; Hoffman la Roche, Neuilly sur-Seine, France; 10 mg/kg, i.p.) as needed. Control animals received saline in place of pilocarpine. Animals were video-monitored 8 h a day for general behavior and occurrence of spontaneous seizures by four weeks after SE (Figure 1). We classified chronic epilepsy rats that showed behavioral seizures with seizure score ≥ 3 more than once.

### 2.3. Surgery

Control and epilepsy rats were implanted with monopolar stainless steel electrodes (Plastics One, Roanoke, VA, USA) in the right hippocampus (stereotaxic coordinates was −3.8 mm posterior; 2.0 mm lateral; −2.6 mm depth to bregma) under isoflurane anesthesia (3% induction, 1.5−2% for surgery, and 1.5% maintenance in a 65:35 mixture of N_2_O:O_2_). Some animals were also implanted with a brain infusion kit 1 (Alzet, Cupertino, CA, USA) to infuse with vehicle or cyclosporin A (CsA, a PP2B inhibitor, 250 μM) into the right lateral ventricle (1 mm posterior; 1.5 mm lateral; −3.5 mm depth to the bregma, see below). The CsA concentration did not affect spontaneous seizure activities in chronic epilepsy rats [30]. Throughout surgery, the core temperature of each rat was maintained at 37–38 °C. Electrodes were secured to the exposed skull with dental acrylic. 

### 2.4. Drug Trials, EEG Analysis and Quantification of Behavioral Seizure Activity

#### 2.4.1. Experiment I

Figure 1 illustrates the design of the drug trial methodology, which was a modified protocol based on previous studies [10,26,30,31]. After baseline seizure activity was determined over three days, perampanel (8 mg/kg, i.p, Eisai Korea Inc., Seoul, Korea), GYKI 52466 (10 mg/kg, i.p.) or saline (vehicle) was daily administered at 6:00 PM over a one-week period [4,30]. Electroencephalographic (EEG) signals were detected with a DAM 80 differential amplifier (0.1–3000 Hz bandpass; World Precision Instruments, Sarasota, FL, USA) 2 h a day at the same time over a one-week period. The data were digitized (1000 Hz) and analyzed using LabChart Pro v7 (ADInstruments, Bella Vista, New South Wales, Australia). Behavioral seizure severity was evaluated according to Racine’s scale [32]: 1, immobility, eye closure, twitching of vibrissae, sniffing, facial clonus; 2, head nodding associated with more severe facial clonus; 3, clonus of one forelimb; 4, rearing, often accompanied by bilateral forelimb clonus; and 5, rearing with loss of balance and falling accompanied by generalized clonic seizures. After recording (18 h after the last drug treatment), animals were used for Western blot.

#### 2.4.2. Experiment II

Some non-responders in experiment I were given saline (i.p.) over a seven-day period. Thereafter, perampanel or GYKI 52466 was daily administered by the aforementioned method. Non-responders were also connected with Alzet 1007D osmotic pump (Alzet, Cupertino, CA, USA) containing vehicle or CsA (250 μM). The pump was placed in a subcutaneous pocket in the dorsal region. After recording (18 h after the last drug treatment), animals were used for Western blot.

### 2.5. Co-Immunoprecipitation

The hippocampal tissues were lysed in radioimmunoprecipitation assay buffer (RIPA: 50 mM Tris–HCl pH 8.0; 1% Nonidet P-40; 0.5% deoxycholate; 0.1% SDS, Thermo Fisher Scientific Korea, Seoul, South Korea) containing a protease inhibitor cocktail (Roche Applied Sciences, Branford, CT, USA), phosphatase inhibitor cocktail (PhosSTOP^®^, Roche Applied Science, Branford, CT, USA) and 1 mM sodium orthovanadate. Protein concentrations were calibrated by BCA protein assay (Pierce Chemical, Rockford, IL, USA) and equal amounts of total proteins were incubated with NEDD4-2, SGK1 or GluA1 antibody (Table 1) and protein G sepharose beads at 4 °C overnight. Beads were collected by centrifugation, eluted in 2× SDS sample buffer, and boiled at 95 °C for 5 min. Thereafter, Western blots for ubiquitin were performed.

### 2.6. Western Blot

Animals were sacrificed by decapitation, and their hippocampi were obtained and homogenized in lysis buffer containing protease inhibitor cocktail (Roche Applied Sciences, Branford, CT, USA) and phosphatase inhibitor cocktail (PhosSTOP^®^, Roche Applied Science, Branford, CT, USA). Thereafter, total protein concentration was calibrated using a Micro BCA Protein Assay Kit (Pierce Chemical, Rockford, IL, USA). Western blot was performed by the standard protocol: Sample proteins (10 μg) were separated on a Bis-Tris sodium dodecyl sulfate-poly-acrylamide gel (SDS-PAGE) and transferred to membranes. Membranes were incubated with 2% bovine serum albumin (BSA) in Tris-buffered saline (TBS; in mM 10 Tris, 150 NaCl, pH 7.5, and 0.05% Tween 20), and then reacted with primary antibodies (Table 1) overnight at 4 °C. After washing, membranes were incubated in a solution containing horseradish peroxidase (HRP)-conjugated secondary antibodies for 1 h at room temperature. Immunoblots were detected and quantified using an ImageQuant LAS4000 system (GE Healthcare Korea, Seoul, Korea). Optical densities of proteins were calculated with the corresponding amount of β-actin.

### 2.7. Data Analysis

The Shapiro–Wilk *W*-test was used to evaluate the normality values. Mann–Whitney U-test, Wilcoxon signed rank test, Student’s *t*-test, and paired Student’s *t*-test were applied to determine statistical significance of data. Comparisons among groups were also performed using repeated measures ANOVA, Friedman test and one-way ANOVA followed by Bonferroni’s post hoc comparisons. A *p*-value less than 0.05 was considered to be significant.

## 3. Results

### 3.1. AMPAR Antagonists Attenuate Spontaneous Seizure Activity in Responders

**Figure 2:**
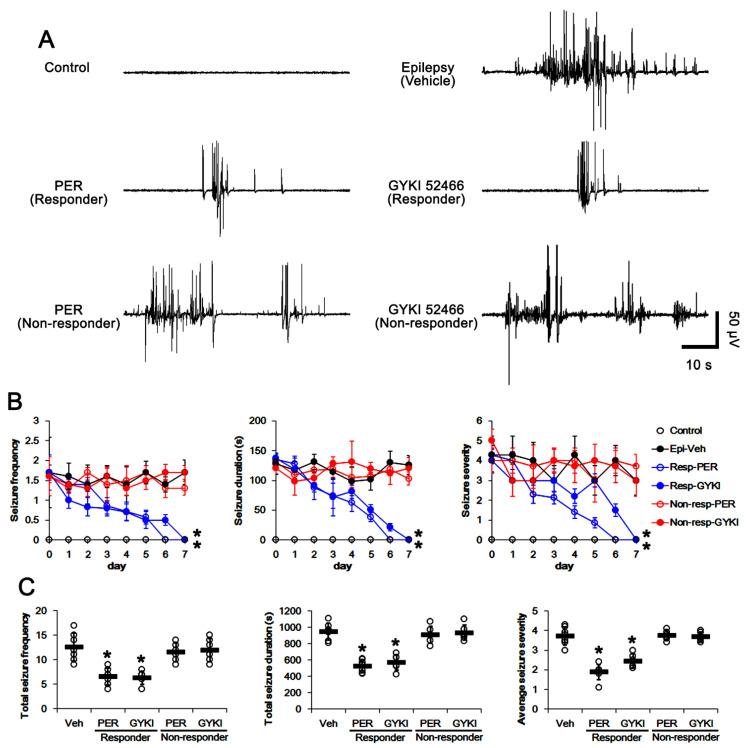
The effects of perampanel (PER) and GYKI 52466 (GYKI) on spontaneous seizure activities in chronic epilepsy rats. Both α-amino-3-hydroxy-5-methylisoxazole-4-propionic acid receptor (AMPAR) antagonists effectively attenuate spontaneous seizure activities in responders. (**A**) Representative electroencephalograms (EEG) in each group at 2 days after treatment. (**B**) Quantitative analyses of the chronological effects of AMPAR antagonists on seizure frequency, seizure duration and seizure severity (seizure score) over a seven-day period. Error bars indicate SD (* p < 0.05 vs. vehicle (Veh)-treated animals; Friedman test for seizure frequency and seizure severity; repeated measures ANOVA for seizure duration). (**C**) Quantitative analyses of seizure frequency, total seizure duration and average behavioral seizure score (seizure severity) over a seven-day period. Open circles indicate each individual value. Horizontal bars indicate mean value. Error bars indicate SD (* p < 0.05 vs. vehicle (Veh)-treated animals; Mann–Whitney U-test for seizure frequency and seizure severity; Student *t*-test for seizure duration).

In epileptic rats, the total seizure frequency (number of seizures), the total electroencephalographic (EEG) seizure duration and average seizure severity (behavioral seizure core) were 12.6 ± 2.9, 945.8 ± 102 s and 3.7 ± 0.5 over a one-week period, respectively (*n* = 7, Figure 2A–C). In responders (showing the significant reduction in seizure activities), perampanel gradually reduced seizure frequency (*χ^2^*_(1)_ = 5.1, *p* = 0.024, Friedman test, *n* = 7), seizure duration (*F*_(1,12)_ = 6.8, *p* = 0.022, repeated measures ANOVA, *n* = 7) and the seizure severity (*χ^2^*_(1)_ = 5.6, *p* = 0.018 Friedman test, *n* = 7) over a one-week period (Figure 2A,B). The total seizure frequency was 6.57 ± 1.72 (*z* = 3.07, *p* = 0.002 vs. vehicle, Mann–Whitney U-test, *n* = 7), the total seizure duration was 538.3 ± 127 s (*t*_(12)_ = 8.54, *p* < 0.001 vs. vehicle, Student *t*-test, *n* = 7), and the average seizure severity was 1.9 ± 0.4 over a one-week period (*z* = 3.14, *p* = 0.002 vs. vehicle, Mann–Whitney U-test, *n* = 7; Figure 2C). Six out of thirteen rats in the perampanel-treated group were identified as non-responders whose seizure activities were uncontrolled by perampanel (total seizure frequency, 11.5 ± 1.9; total seizure duration, 909 ± 103.9 s; average seizure severity, 3.8 ± 0.2; Figure 2B,C). 

GYKI 52466 also decreased seizure frequency (*χ^2^*_(1)_ = 4.6, *p* = 0.033, Friedman test, *n* = 6), total seizure duration (*F*_(1,11)_ = 5.9, *p* = 0.033, repeated measures ANOVA, *n* = 6), and seizure severity (*χ^2^*_(1)_ = 4.7, *p* = 0.031, Friedman test, *n* = 6) in responders over a one-week period (Figure 2A,B). In responders to GYKI 52466, the total seizure frequency was 6.3 ± 1.4 (*z* = 3.01, *p* = 0.001 vs. vehicle, Mann–Whitney U-test, *n* = 6), the total seizure duration was 571.2 ± 94.1 s (*t*_(11)_ = 6.82, *p* < 0.001 vs. vehicle, Student *t*-test, *n* = 6), and the average seizure severity was 2.5 ± 0.4 over a one-week period (*z* = 2.94, *p* = 0.003 vs. vehicle, Mann–Whitney U-test, *n* = 6; Figure 2C). Six out of twelve rats in the GYKI 52466-treated group were identified as non-responders (total seizure frequency, 12 ± 2.4; total seizure duration, 935.2 ± 95.3 s; average seizure severity, 3.7 ± 0.2; Figure 2B,C). 

### 3.2. AMPAR Antagonists Facilitates NEDD4-2-Mediated GRIA1 Ubiquitination by Enhancing NEDD4-2 S448 Phosphorylation in Responders

NEDD4-2 plays an important role in the regulation of seizure susceptibility, and its phosphorylation level is closely related to the maintenance of its stability, which modulates AMPAR functionality [17,33]. Thus, we explored whether AMPAR antagonists affect NEDD4-2 protein expression and its phosphorylation levels.

**Figure 3:**
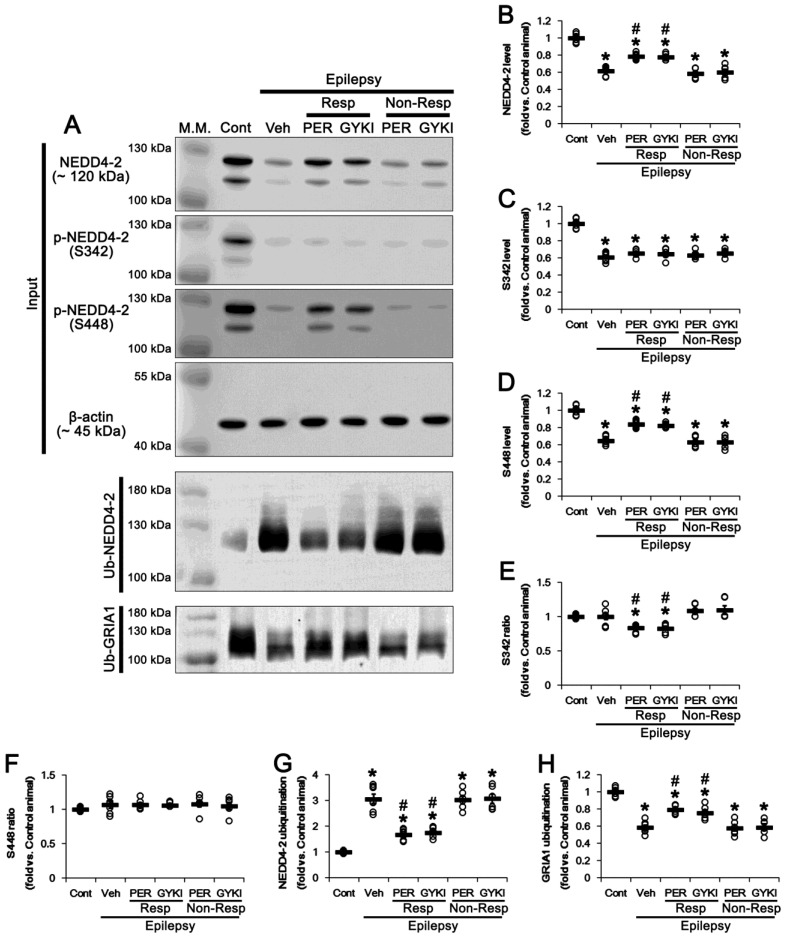
The effects of perampanel (PER) and GYKI 52466 (GYKI) on total NEDD4-2 protein expression/phosphorylation and ubiquitination of NEDD4-2 and GRIA1. (**A**) Representative images for Western blot of NEDD4-2 protein expression/phosphorylation and ubiquitination of NEDD4-2 and GRIA1 in the hippocampal tissues. (**B**–**F**) Quantifications of NEDD4-2 level (**B**), p-NEDD4-2 S342 level (**C**), p-NEDD4-2 S448 level (**D**), p-NEDD4-2 S342/NEDD4-2 ratio (**E**) and p-NEDD4-2 S448/NEDD4-2 ratio (**F**) in the hippocampal tissues. (**G**–**H**) Quantifications of the bindings of NEDD4-2 (**G**) and GRIA1 (**H**) with ubiquitin (Ub) in the hippocampal tissues. Open circles indicate each individual value. Horizontal bars indicate mean value. Error bars indicate SEM (*,# *p* < 0.05 vs. control and vehicle (Veh)-treated animals, respectively; one-way ANOVA with post hoc Bonferroni’s multiple comparison).

Consistent with previous studies [34], NEDD4-2 protein level was 39% lower in the epileptic hippocampus (*t*_(12)_ = 14.2, *p* < 0.001 vs. control animals, Student *t*-test; Figure 3A,B and Figure S1), as compared to control animals. Compatible with its protein level, NEDD4-2 S342 and NEDD4-2 S448 phosphorylation levels were decreased to 0.61 (*t*_(12)_ = 13.3, *p* < 0.001 vs. control animals, Student *t*-test) and 0.65 times (*t*_(12)_ = 12.9, *p* < 0.001 vs. control animals, Student *t*-test) the control level in the vehicle-treated epilepsy rats, respectively (Figure 3A,C,D and Figure S1). The NEDD4-2 S342 and S448 phosphorylation ratios in epilepsy rats were similar to those in control animals (Figure 3E,F and Figure S1).

In responders to perampanel and GYKI 52466, NEDD4-2 protein levels were increased to 0.78 and 0.77 times the control level, respectively (*F*_(2,17)_ = 35.8, *p* < 0.001 vs. vehicle, one-way ANOVA; Figure 3A,B and Figure S1). NEDD4-2 S342 phosphorylation level was unaffected by both AMPAR antagonists (*F*_(2,17)_ = 1.4, *p* = 0.28 vs. vehicle, one-way ANOVA; Figure 3A,C and Figure S1). Due to upregulation of NEDD4-2 protein level induced by AMPAR antagonists, S342 phosphorylation ratios were reduced to 0.84 and 0.83 times the control level in perampanel and GYKI 52466-treated epilepsy rats, respectively (*F*_(2,17)_ = 8.3, *p* = 0.003 vs. vehicle, one-way ANOVA; Figure 3A, E and Figure S1). However, perampanel and GYKI 52466 increased NEDD4-2 S448 phosphorylation level to 0.84 and 0.83 times the control level, respectively (*F*_(2,17)_ = 47.9, *p* < 0.001 vs. vehicle, one-way ANOVA; Figure 3A,D). In contrast to NEDD4-2 S342 phosphorylation ratio, neither AMPAR antagonist altered NEDD4-2 S448 phosphorylation ratios (*F*_(2,17)_ = 0.01, *p* = 0.99 vs. vehicle, one-way ANOVA; Figure 3A,F and Figure S1). In non-responders to perampanel and GYKI 52466, NEDD4-2 protein level and its S342 and S448 phosphorylation levels/ratios were unchanged by each compound (Figure 3A–F and Figure S1). Since phosphorylation stabilize NEDD4-2 against ubiquitination [17,35], we also investigated the effects of AMPAR antagonists on NEDD4-2 ubiquitination. In epileptic hippocampuses, NEDD4-2 ubiquitination (ubiquitin (Ub)-NEDD4-2 binding) was increased to 3.07 times the control level (*t*_(12)_ = 11.2, *p* < 0.001 vs. control animals, Student *t*-test; Figure 3A,G and Figure S1), while GRIA1 ubiquitination was reduced to 0.58 times the control level (*t*_(12)_ = 13.1, *p* < 0.001 vs. control animals, Student *t*-test; Figure 3A,H). In responders to perampanel and GYKI 52466, NEDD4-2 ubiquitination was reduced to 1.67 and 1.76 times the control level, respectively (*F*_(2,17)_ = 38.8, *p* < 0.001 vs. vehicle, one-way ANOVA; Figure 3A,G and Figure S1). In contrast, GRIA1 ubiquitination was increased to 0.8 and 0.76 times the control level, respectively (*F*_(2,17)_ = 20.7, *p* < 0.001 vs. vehicle, one-way ANOVA; Figure 3A,H and Figure S1). In non-responders to perampanel and GYKI 52466, ubiquitination of NEDD4-2 and GRIA1 were unaffected by each AMPAR antagonist (Figure 3A,G,H and Figure S1). Therefore, our findings indicate that the regulation of NEDD4-2-mediated GRIA1 ubiquitination may be relevant to the responsiveness to AMPAR antagonists that inhibit spontaneous seizure activity.

### 3.3. AMPAR Antagonists Enhance SGK1 S78 Phosphorylation, but Not Protein Level, in Responders

Serum and glucocorticoid-regulated kinase 1 (SGK1) plays a key role in NEDD4-2 phosphorylation, and SGK1 and NEDD4-2 regulate one another in a reciprocal manner: SGK1-mediated NEDD4-2 phosphorylation increases SGK1 ubiquitination by NEDD4-2 [36]. Therefore, we investigated the effects of AMPAR antagonists on SGK1-NEDD4-2 interactions in chronic epilepsy rats. 

**Figure 4:**
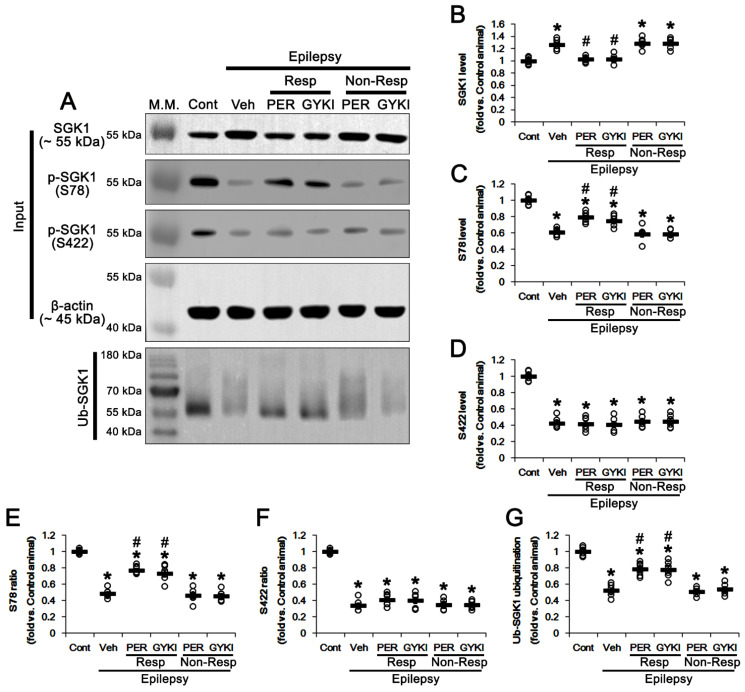
The effects of perampanel (PER) and GYKI 52466 (GYKI) on total SGK1 protein expression/phosphorylation and SGK1 ubiquitination. (**A**) Representative images for Western blot of SGK1 protein expression/phosphorylation and SGK1 ubiquitination in the hippocampal tissues. (**B**–**F**) Quantifications of SGK1 level (**B**), p-SGK1 S78 level (**C**), p-SGK1 S422 level (**D**), p-SGK1 S78/SGK1 ratio (**E**) and p-SGK1 S422/SGK1 ratio (**F**) in the hippocampal tissues. (**G**) Quantifications of the binding of SGK1 with ubiquitin (Ub) in the hippocampal tissues. Open circles indicate each individual value. Horizontal bars indicate mean value. Error bars indicate SEM (*,# *p* < 0.05 vs. control and vehicle (Veh)-treated animals, respectively; one-way ANOVA with post hoc Bonferroni’s multiple comparison).

In the epileptic hippocampus, SGK1 protein level was increased to 1.26 times the control level (*t*_(12)_ = 7.5, *p* < 0.001 vs. control animals, Student *t*-test; Figure 4A,B and Figure S2). SGK1 S78 and S422 phosphorylation levels were decreased to 0.61 (*t*_(12)_ = 14.9, *p* < 0.001 vs. control animals, Student *t*-test) and 0.43 times (*t*_(12)_ = 18.5, *p* < 0.001 vs. control animals, Student *t*-test) the control level in epilepsy rats, respectively (Figure 4A,C,D). The SGK1 S78 and S422 phosphorylation ratios in epilepsy rats were reduced to 0.48 (*t*_(12)_ = 24.3, *p* < 0.001 vs. control animals, Student *t*-test) and 0.34 times (*t*_(12)_ = 25.8, *p* < 0.001 vs. control animals, Student *t*-test) the control level (Figure 4E,F and Figure S2).

In responders, both AMPAR antagonists restored SGK1 protein levels to control level (*F*_(2,17)_ = 29.7, *p* < 0.001 vs. vehicle, one-way ANOVA; Figure 4A,B). Perampanel and GYKI 52466 also increased SGK1 S78 phosphorylation level to 0.79 and 0.75 times the control level, respectively (*F*_(2,17)_ = 18.6, *p* < 0.001 vs. vehicle, one-way ANOVA; Figure 4A,C and Figure S2). The SGK1 S78 phosphorylation ratios were increased to 0.77- and 0.73 times the control level in perampanel and GYKI 52466-treated animals, respectively (*F*_(2,17)_ = 36.5, *p* < 0.001 vs. vehicle, one-way ANOVA; Figure 4A,E). However, neither AMPAR antagonist affected the S422 phosphorylation level (*F*_(2,17)_ = 0.1, *p* = 0.92 vs. vehicle, one-way ANOVA; Figure 4A,C,D and Figure S2) or its phosphorylation ratio (*F*_(2,17)_ = 1.7, *p* = 0.22 vs. vehicle, one-way ANOVA; Figure 4E,F and Figure S2). 

Unlike NEDD4-2, SGK1 ubiquitination was reduced to 0.52 times the control level in the epileptic hippocampus (*t*_(12)_ = 14.4, *p* < 0.001 vs. control animals, Student *t*-test; Figure 4A,G). In responders to perampanel and GYKI 52466, SGK1 ubiquitination were 0.78 and 0.77 times the control level, respectively (*F*_(2,17)_ = 19.6, *p* < 0.001 vs. vehicle, one-way ANOVA; Figure 4A,G and Figure S2). In non-responders to perampanel and GYKI 52466, SGK1 protein level, its S78 and S422 phosphorylation levels/ratios, and SGK1 ubiquitination were unaffected by each compound (Figure 4A–G and Figure S2). These findings indicate that AMPAR antagonists may selectively enhance SGK1 78 phosphorylation, which phosphorylates NEDD4-2 S448 site. Furthermore, considering NEDD4-2-mediated SGK1 ubiquitination [36], it is likely that upregulation of NEDD4-2 phosphorylation induced by AMPAR antagonists will lead to SGK1 ubiquitination. 

### 3.4. AMPAR Antagonists Increases ERK1/2, but Reduces PDK1, Phosphorylation in Responders

Mitogen-activated protein kinase (MAPK)/extracellular signal-regulated kinase 1/2 (ERK1/2) and phosphatidylinositol 3-kinases (PI3K)/3-phosphoinositide-dependent protein kinase-1 (PDK1)/AKT pathways phosphorylate SGK1 at S78 and S422 sites, respectively [37,38,39,40]. In addition, PI3K/PDK1/AKT cascade directly phosphorylates NEDD4-2 [41]. Thus, we explored the effects of AMPAR antagonists on PDK1 and AKT activities (phosphorylation). 

**Figure 5:**
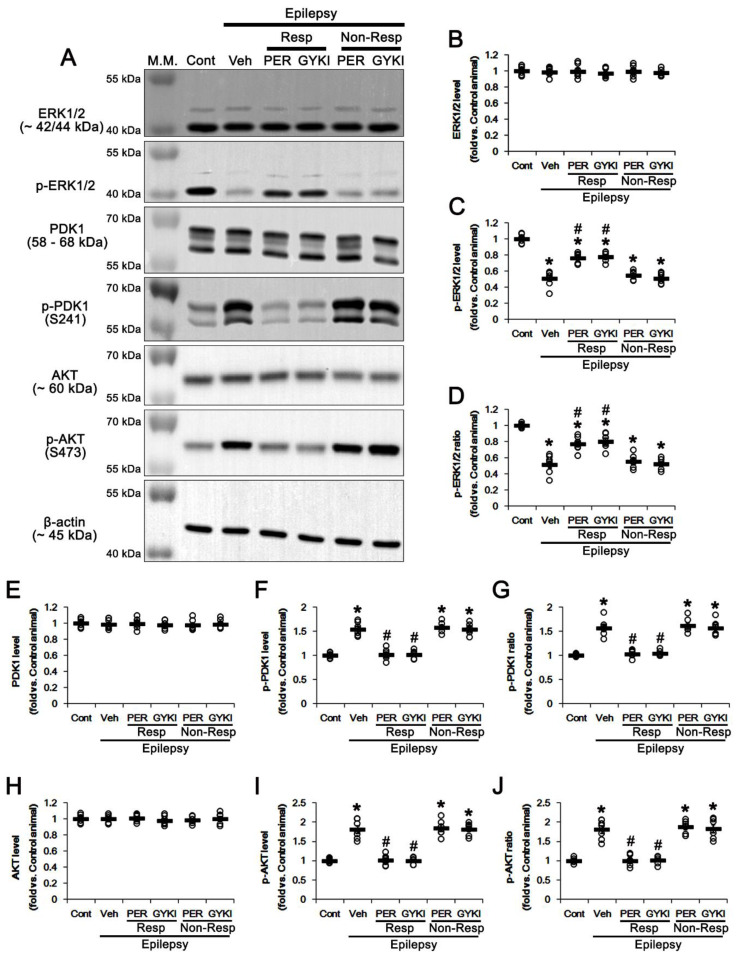
The effects of perampanel (PER) and GYKI 52466 (GYKI) on protein and phosphorylation levels of ERK1/2, PDK1 and AKT. (**A**) Representative images for Western blot of protein and phosphorylation levels of ERK1/2, PDK1 and AKT. (**B**–**J**) Quantifications of ERK1/2 level (**B**), p-ERK1/2 level (**C**), p-ERK1/2 ratio (**D**), PDK1 level (**E**), p-PDK1 level (**F**), p-PDK1 ratio (**G**), AKT level (**H**), p-AKT level (**I**) and p-AKT ratio (**J**) in the hippocampal tissues. Open circles indicate each individual value. Horizontal bars indicate mean value. Error bars indicate SEM (*,# *p* < 0.05 vs. control and vehicle (Veh)-treated animals, respectively; one-way ANOVA with post hoc Bonferroni’s multiple comparison).

In the present study, ERK1/2 phosphorylation level and its ratio were decreased to 0.51 (*t*_(12)_ = 11.6, *p* < 0.001 vs. control animals, Student *t*-test) and 0.52 times (*t*_(12)_ = 10.9, *p* < 0.001 vs. control animals, Student *t*-test) the control level in epilepsy rats, respectively, without altering ERK1/2 protein level (Figure 5A–D and Figure S3). In contrast, PDK1 phosphorylation level and its ratio were increased to 1.55 (*t*_(12)_ = 10.0, *p* < 0.001 vs. control animals, Student *t*-test) and 1.57 times (*t*_(12)_ = 8.7, *p* < 0.001 vs. control animals, Student *t*-test) the control level in epilepsy rats, respectively, while PDK1 protein level was unchanged (Figure 5A,E–G and Figure S3). AKT phosphorylation level and its ratio were also increased to 1.8 (*t*_(12)_ = 8.9, *p* < 0.001 vs. control animals, Student *t*-test) and 1.81 times (*t*_(12)_ = 8.4, *p* < 0.001 vs. control animals, Student *t*-test) the control level in epilepsy rats, respectively, without changing AKT protein level (Figure 5A,H–J and Figure S3).

In responders, neither AMPAR antagonist affected ERK1/2 protein levels (*F*_(2,17)_ = 0.3, *p* = 0.78 vs. vehicle, one-way ANOVA; Figure 5A,B and Figure S3). However, perampanel and GYKI 52466 enhanced ERK1/2 phosphorylation level to 0.76 and 0.78 times the control level, respectively (*F*_(2,17)_ = 26.9, *p* < 0.001 vs. vehicle, one-way ANOVA; Figure 5A,C and Figure S3). The ERK1/2 phosphorylation ratios were also increased to 0.77 and 0.8 times the control level, respectively (*F*_(2,17)_ = 15.6, *p* < 0.001 vs. vehicle, one-way ANOVA; Figure 5A,D and Figure S3). In contrast to ERK1/2, both AMPAR antagonists restored PDK1 phosphorylation level (*F*_(2,17)_ = 50.5, *p* < 0.001 vs. vehicle, one-way ANOVA), PDK1 phosphorylation ratio (*F*_(2,17)_ = 49.6, *p* < 0.001 vs. vehicle, one-way ANOVA), AKT phosphorylation level (*F*_(2,17)_ = 52, *p* < 0.001 vs. vehicle, one-way ANOVA) and AKT phosphorylation ratio (*F*_(2,17)_ = 45.4, *p* < 0.001 vs. vehicle, one-way ANOVA) to control level without affecting their protein levels (Figure 5A,E–J and Figure S3). In non-responders, perampanel and GYKI 52466 did not result in these phenomena (Figure 5A–J and Figure S3). These findings indicate that AMPAR antagonists may increase NEDD4-2 S448 phosphorylation via ERK1/2-mediated SGK1 activation, independent of PI3K/PDK1/AKT signaling pathway. 

### 3.5. Effects of AMPAR Antagonists on Protein Phosphatase Phosphorylation

Protein phosphatase 2A (PP2A) and protein phosphatase 2B (PP2B) inhibit ERK1/2 kinase activity by dephosphorylating threonine and tyrosine residues [42,43]. In our previous study [30], perampanel increases PP2B, but not PP2A, phosphorylation ratio (inactivation), while their expressions/phosphorylation ratios in epileptic animals are lower than those in normal animals. Since PP2A leads to SGK1 inactivation [44], we confirmed whether both AMPAR antagonists activate ERK1/2-medaited SGK1 S78 phosphorylation by inhibiting PP2B, but not PP2A.

**Figure 6:**
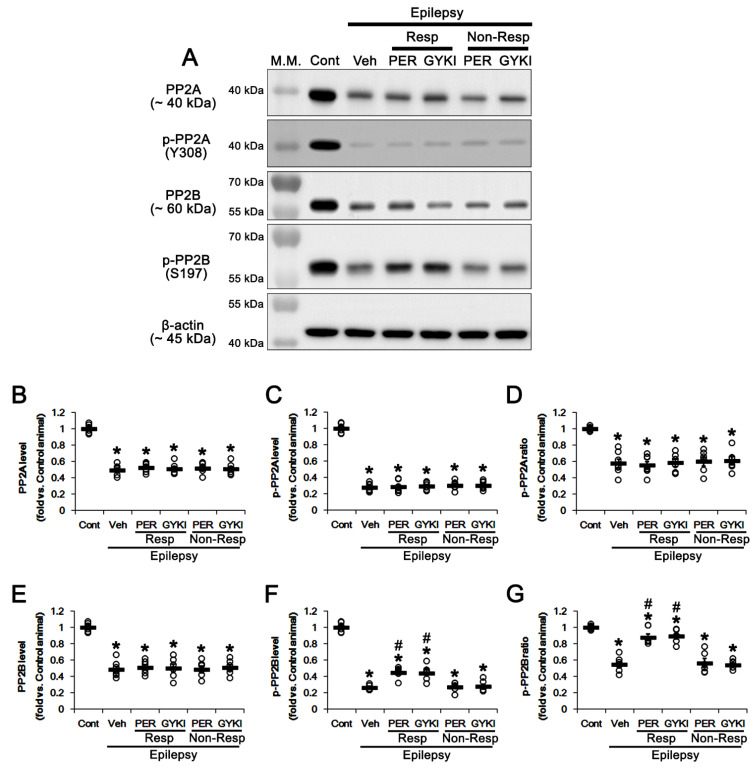
The effects of perampanel (PER) and GYKI 52466 (GYKI) on protein and phosphorylation levels of PP2A and PP2B. (**A**) Representative images for Western blot of protein and phosphorylation levels of PP2A and PP2B. (**B**–**G**) Quantifications of PP2A level (**B**), p-PP2A level (**C**), p-PP2A ratio (**D**), PP2B level (**E**), p-PP2B level (**F**) and p-PP2B ratio (**G**). Open circles indicate each individual value. Horizontal bars indicate mean value. Error bars indicate SEM (*,# *p* < 0.05 vs. control and vehicle (Veh)-treated animals, respectively; one-way ANOVA with post hoc Bonferroni’s multiple comparison).

Consistent with our previous study [30], the present study showed that PP2A protein level was 0.5 times the control level in the epileptic hippocampus (*t*_(12)_ = 16.3, *p* < 0.001 vs. control animals, Student *t*-test; Figure 6A,B and Figure S4). PP2A phosphorylation level and its ratio were 0.28 (*t*_(12)_ = 26, *p* < 0.001 vs. control animals, Student *t*-test) and 0.58 times (*t*_(12)_ = 8, *p* < 0.001 vs. control animals, Student *t*-test) the control level in epilepsy rats, respectively (Figure 6A,C,D and Figure S4). Similarly, PP2B protein level was reduced to 0.49 times the control level in the epileptic hippocampus (*t*_(12)_ = 12.6, *p* < 0.001 vs. control animals, Student *t*-test; Figure 6A,E and Figure S4). PP2B phosphorylation level and its ratio were decreased to 0.26 (*t*_(12)_ = 30.3, *p* < 0.001 vs. control animals, Student *t*-test) and 0.55 times (*t*_(12)_ = 12.9, *p* < 0.001 vs. control animals, Student *t*-test) the control level in epilepsy rats, respectively (Figure 6A,F,G and Figure S4). Since phosphorylation negatively regulates PP2A and PP2B activities [45,46], these findings indicate that PP2A and PP2B activities may be enhanced in the epileptic hippocampus as compensatory responses to downregulation of their protein levels. 

In responders, neither AMPAR antagonist affected PP2A protein levels (*F*_(2,17)_ = 0.3, *p* = 0.73 vs. vehicle, one-way ANOVA), PP2A phosphorylation levels (*F*_(2,17)_ = 0.1, *p* = 0.93 vs. vehicle, one-way ANOVA) or its phosphorylation ratio (*F*_(2,17)_ = 0.11, *p* = 0.89 vs. vehicle, one-way ANOVA; Figure 6A–D and Figure S4). In contrast, perampanel and GYKI 52466 increased PP2B phosphorylation level to 0.45 and 0.44 times the control level (*F*_(2,17)_ = 17.2, *p* < 0.001 vs. vehicle, one-way ANOVA), respectively, without altering its protein level (Figure 6A,E,F and Figure S4). Thus, PP2B phosphorylation ratios were enhanced to 0.88 and 0.89 times the control level in perampanel- and GYKI 52466-treated animals, respectively (*F*_(2,17)_ = 32.7, *p* < 0.001 vs. vehicle, one-way ANOVA; Figure 6A,G and Figure S4). In non-responders, perampanel and GYKI 52466 did not affect the protein and phosphorylation levels of these phosphatases (Figure 6A–G and Figure S4). These findings indicate that AMPAR antagonists may increase PP2B, but not PP2A, phosphorylation (inactivation), which would affect the ERK1/2-SGK1-NEDD4-2 signaling pathway.

### 3.6. Co-Treatment of PP2B Inhibitor Increases the Efficacies of AMPAR Antagonists in Non-Responders

**Figure 7:**
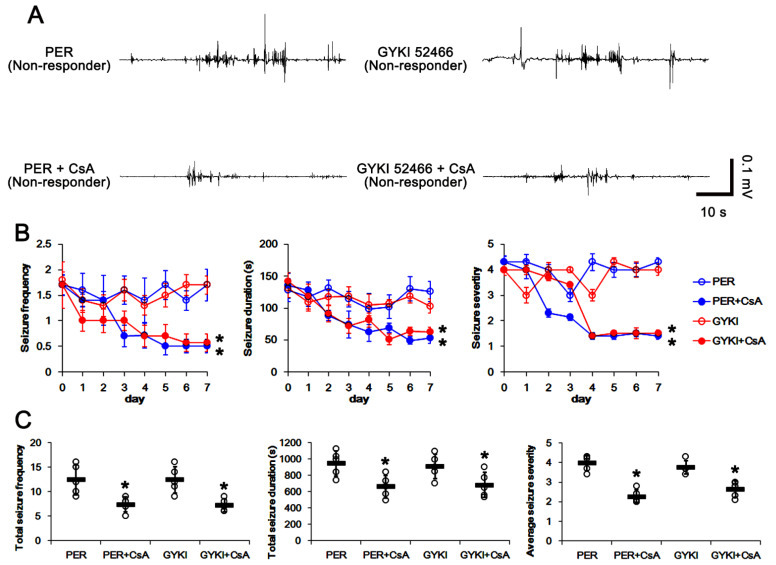
The effects of CsA co-treatment with perampanel (PER) and GYKI 52466 (GYKI) on spontaneous seizure activities in non-responders. CsA co-treatment effectively improves the anti-epileptic effects of both AMPAR antagonists in non-responders. (**A**) Representative electroencephalograms (EEG) in each group at two days after CsA co-treatment. (**B**) Quantitative analyses of the chronological effects of CsA co-treatment withAMPAR antagonists on seizure frequency, seizure duration and seizure severity (seizure score) over seven-day period. Error bars indicate SD (* p < 0.05 vs. vehicle (Veh)-treated animals; Friedman test for seizure frequency and seizure severity; repeated measures ANOVA for seizure duration). (**C**) Quantitative analyses of seizure frequency, total seizure duration and average behavioral seizure score (seizure severity) in seven-day period. Open circles indicate each individual value. Horizontal bars indicate mean value. Error bars indicate SD (* p < 0.05 vs. vehicle (Veh)-treated animals; Wilcoxon signed rank test for seizure frequency and seizure severity; paired Student *t*-test for seizure duration).

To confirm the role of the PP2B-ERK1/2-SGK1-NEDD4-2 signaling pathway in GRIA1 ubiquitination and refractory seizures to AMPAR antagonists, cyclosporin A (CsA, a PP2B inhibitor) was co-treated with perampanel or GYKI 52466 in non-responders. In non-responders to perampanel, total seizure frequency was 12.4 ± 3.1, total seizure duration was 946.4 ± 153.1 s, and average seizure severity was 4 ± 0.4 over a one-week period (*n* = 5, Figure 7A–C). In non-responders to GYKI 52466, total seizure frequency was 12 ± 2.7, total seizure duration was 908.8 ± 146.9 s, and average seizure severity was 3.8 ± 0.3 over a one-week period (*n* = 5, Figure 7A–C). 

CsA co-treatment gradually decreased seizure frequency (*χ^2^*_(3)_ = 8.4, *p* = 0.038, Friedman test, *n* = 5), total seizure duration (*F*_(3,16)_ = 4.2, *p* = 0.023, repeated measures ANOVA, *n* = 5), and seizure severity (*χ^2^*_(3)_ = 9.7, *p* = 0.021, Friedman test, *n* = 5) in both perampanel- and GYKI 52466-treated groups over a one-week period (Figure 7A,B). In non-responders to perampanel, CsA co-treatment reduced the total seizure frequency to 7.4 ± 1.5 (*z* = 2.02, *p* = 0.043, Wilcoxon signed rank test, *n* = 5; Figure 7A,C), the total seizure duration to 660 ± 136.8 s (*t*_(4)_ = 3.58, *p* = 0.02, paired Student *t*-test, *n* = 5; Figure 7A,C), and average seizure severity to 2.3 ± 0.3 (*z* = 2.03, *p* = 0.042, Wilcoxon signed rank test, *n* = 5; Figure 7A,C). In non-responders to GYKI 52466, CsA co-treatment also attenuated the seizure frequency to 7.5 ± 1.3 (*z* = 2.04, *p* = 0.041, Wilcoxon signed rank test, *n* = 5; Figure 7A,C), the total seizure duration to 680.8 ± 153.1 s (*t*_(4)_ = 3.2, *p* = 0.03, paired Student *t*-test, *n* = 5; Figure 7A,C), and the seizure severity to 2.6 ± 0.4 (*z* = 2.02, *p* = 0.043 vs. vehicle, Wilcoxon signed rank test, *n* = 5; Figure 7A,C). Taken together, these findings indicate that dysregulation of the PP2B-ERK1/2-SGK1-NEDD4-2 signaling pathway may play an important role in the generation of refractory seizures to AMPAR antagonists.

### 3.7. CsA Co-Treatment Facilicates GRIA1 Ubiquitination in Non-Responders to AMPAR Antagonists

Next, we investigated whether CsA co-treatment influences ERK1/2- or AKT-mediated SGK1-NEDD4-2 regulation in non-responders to AMPAR antagonists.

**Figure 8:**
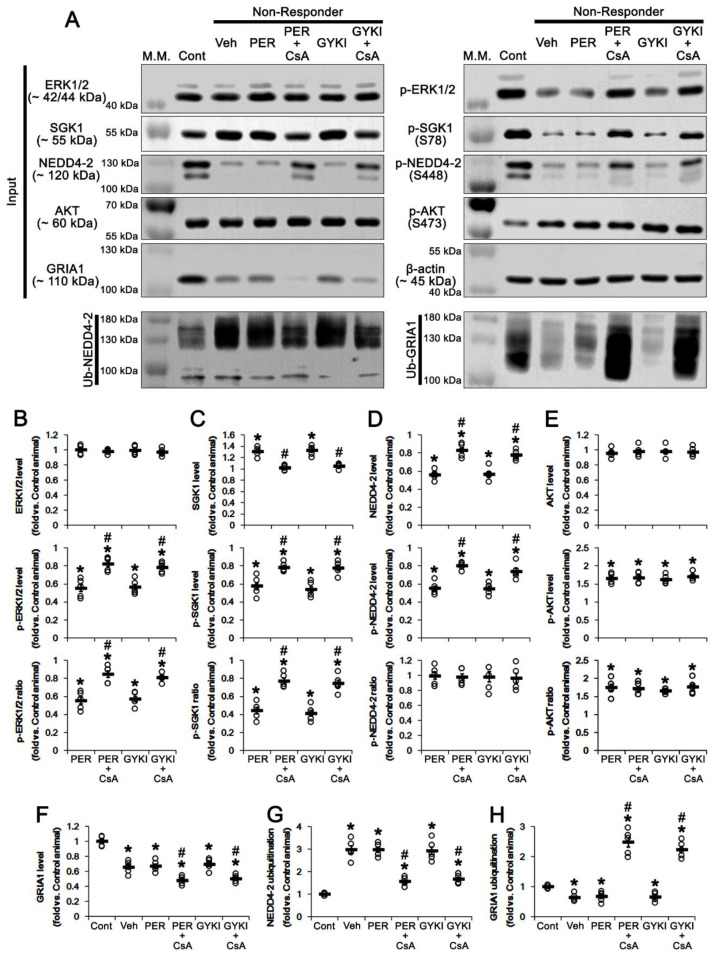
The effects of CsA co-treatment with perampanel (PER) and GYKI 52466 (GYKI) on expression/phosphorylation levels of ERK1/2, SGK1, NEDD4-2 and AKT, and ubiquitination of NEDD4-2 and GRIA1. (**A**) Representative images for Western blot of ERK1/2, SGK1, NEDD4-2 and AKT and ubiquitination of NEDD4-2 and GRIA1 in the hippocampal tissues. (**B**–**E**) Quantifications of protein level, phosphorylation level and phosphorylation ratio of ERK1/2 (**B**), SGK1 (**C**), NEDD4-2 (**D**) and AKT (**E**) in the hippocampal tissues. (**F**–**H**) Quantifications of GRIA1 level (**F**) and the bindings of NEDD4-2 (**G**) and GRIA1 (**H**) with ubiquitin (Ub) in the hippocampal tissues. Open circles indicate each individual value. Horizontal bars indicate mean value. Error bars indicate SEM (*,# *p* < 0.05 vs. control and vehicle (Veh)-treated animals, respectively; one-way ANOVA with post hoc Bonferroni’s multiple comparison).

CsA co-treatment did not affect ERK1/2 protein level in non-responders (Figure 8A,B). However, CsA co-treatment enhanced ERK1/2 phosphorylation to 0.82 and 0.78 times the control level in non-responders to perampanel (*t*_(8)_ = 4.73, *p* = 0.001, Student *t*-test) and GYKI 52466 (*t*_(8)_ = 4.9, *p* = 0.001, Student *t*-test), respectively (Figure 8A,B and Figure S5). Thus, CsA co-treatment increased ERK1/2 phosphorylation ratio in non-responders to perampanel (*t*_(8)_ = 4.67, *p* = 0.002, Student *t*-test) and GYKI 52466 (*t*_(8)_ = 5.88, *p* < 0.001, Student *t*-test), respectively (Figure 8A,B and Figure S5). CsA co-treatment restored the increased SGK1 protein level to control level in non-responders to perampanel (*t*_(8)_ = 6.8, *p* < 0.001, Student *t*-test) and GYKI 52466 (*t*_(8)_ = 6.2, *p* < 0.001, Student *t*-test), respectively (Figure 8A,C and Figure S5). However, CsA co-treatment enhanced SGK1 S78 phosphorylation to 0.78 and 0.77 times the control level in non-responders to perampanel (*t*_(8)_ = 4, *p* = 0.004, Student *t*-test) and GYKI 52466 (*t*_(8)_ = 4.8, *p* = 0.001, Student *t*-test), respectively (Figure 8A,C and Figure S5). CsA co-treatment increased the SGK1 S78 phosphorylation ratio to 0.77 and 0.74 times the control level in non-responders to perampanel (*t*_(8)_ = 5.9 *p* < 0.001, Student *t*-test) and GYKI 52466 (*t*_(8)_ = 5.8, *p* < 0.001, Student *t*-test; Figure 8A,C and Figure S5), respectively. In addition, CsA co-treatment increased NEDD4-2 protein level to 0.83- and 0.77 times the control level in non-responders to perampanel (*t*_(8)_ = 6.7, *p* < 0.001, Student *t*-test) and GYKI 52466, respectively (*t*_(8)_ = 5.1, *p* < 0.001, Student *t*-test; Figure 8A,D and Figure S5). CsA co-treatment enhanced NEDD4-2 S448 phosphorylation to 0.8 and 0.74 times the control level in non-responders to perampanel (*t*_(8)_ = 7, *p* < 0.001, Student *t*-test) and GYKI 52466 (*t*_(8)_ = 4, *p* = 0.004, Student *t*-test), respectively (Figure 8A,D and Figure S5). However, CsA co-treatment did not change AKT protein level and its phosphorylation level/ratio (Figure 8A,E and Figure S5). These findings indicate that the PP2B inhibition may improve anti-convulsive effects of AMPAR antagonists in non-responders by regulating ERK1/2-SGK1-NEDD4-2 signaling pathway without affecting AKT activity. 

### 3.8. CsA Co-Treatment Reversely Regulates Ubiquitination of NEDD4-2 and GRIA1 in Non-Responders

Finally, we investigated whether PP2B inhibition affects ubiquitination of NEDD4-2 and GRIA1 in the hippocampus of non-responders, since perampanel reduces total GRIA1 protein level in responders and NEDD4-2 phosphorylation facilitates GRIA1 ubiquitination [4,10,17,26,30].

The present study shows that the total GRIA1 protein level in the epileptic hippocampus was 0.66 times the control level (*t*_(8)_ = 7.3, *p* < 0.001 vs. control animals, Student *t*-test; Figure 8A,F and Figure S5). AMPAR antagonists did not affect GRIA1 protein level in non-responders (Figure 8A,F). CsA co-treatment decreased GRIA1 protein level to 0.48 and 0.5 times the control level in non-responders to perampanel (*t*_(8)_ = 4.5, *p* = 0.002, Student *t*-test) and GYKI 52466 (*t*_(8)_ = 4.7, *p* = 0.001, Student *t*-test), respectively (Figure 8A,F). Consistent with NEDD4-2 S448 phosphorylation, CsA co-treatment also reduced NEDD4-2 ubiquitination to 1.57 and 1.67 times the control level in non-responders to perampanel (*t*_(8)_ = 9.2, *p* < 0.001, Student *t*-test) and GYKI 52466 (*t*_(8)_ = 5.7, *p* = 0.004, Student *t*-test), respectively (Figure 8A,G and Figure S5). GRIA1 ubiquitination in the epileptic hippocampus was 0.64 times the control level (*t*_(8)_ = 5.7, *p* < 0.001 vs. control animals, Student *t*-test; Figure 8A,H and Figure S5). AMPAR antagonists did not affect GRIA1 ubiquitination in non-responders (Figure 8A,H and Figure S5). However, CsA co-treatment increased GRIA1 ubiquitination to 2.48 and 2.23 times the control level in non-responders to perampanel (*t*_(8)_ = 9, *p* < 0.001, Student *t*-test) and GYKI 52466 (*t*_(8)_ = 11.5, *p* < 0.001, Student *t*-test), respectively (Figure 8A,H and Figure S5). These findings indicate that CsA may increase the responsiveness to AMPAR antagonists in non-responders by increasing NEDD4-2-mediated GRIA1 ubiquitination. 

## 4. Discussion

Although the pathogenesis of MTLE has been studied for decades, the underlying mechanisms of intractable MTLE are still elusive. Recently, the dysregulation of ubiquitination has been considered as a potential factor for the generation of refractory epilepsy, since ubiquitination is involved in the modulation of synaptic function [47,48]. Ubiquitination is a posttranslational modification that degrades proteins through a sequential reaction by the ubiquitin-activating enzyme (E1), ubiquitin-conjugating enzyme (E2) and ubiquitin ligase enzyme (E3) [47,48]. Among E3 ubiquitin ligases, NEDD4-2 has been focused on for its dysregulation of cellular trafficking/endocytosis and lysosomal degradation of ion channels and transporters in MTLE [17,34]. Under physiological conditions, NEDD4-2 contributes to the elevation of spontaneous neuronal activity, particularly spontaneous spike frequency, when the AMPAR is activated [11]. Indeed, *NEDD4-2^andi^* mice (in whom one of the major forms of NEDD4-2 in the brain is selectively deficient) are less sensitive to AMPAR activation but very sensitive to AMPAR inhibition. Briefly, the direct AMPAR stimulation by AMPA treatment elevates the synchrony of neuronal activity in wild-type mice more than *NEDD4-2^andi^* mice. In contrast, *NEDD4-2^andi^* mice are very sensitive to 2,3-dihydroxy-6-nitro-7-sulphamoyl-benzo(F)quinoxaline (NBQX, an AMPAR antagonist) with regard to spontaneous spike frequency, although average spontaneous spike amplitude and electrode burst activity do not differ after AMPA or NBQX treatment for either genotype [11]. Paradoxically, *NEDD4-2^andi^* mice show a higher seizure susceptibility than kainic acid, which is recovered by the genetically reducing GRIA1 level [11]. Furthermore, kainic acid-induced seizure activity increases NEDD4-2 ubiquitination without altered GRIA1 surface expression [17]. In addition, the upregulated NEDD4-2 ubiquitination increases the latency of seizure onset and seizure progression in response to kainic acid, accompanied by reduced GRIA1 ubiquitination [17]. Therefore, it is likely that the dysregulated GRIA1/AMPAR-mediated intracellular signaling pathway, rather than the direct AMPAR functionality, may contribute to the dysfunction of NEDD4-2-mediated GRIA1 ubiquitination in the epileptic hippocampus. In the present study, NEDD4-2 protein level was lower in the epileptic hippocampus than that in the normal one, concomitant with decreased phosphorylation levels. AMPAR antagonists effectively increased NEDD4-2 protein and its S448 phosphorylation level in responders, but not in non-responders. Furthermore, AMPAR inhibitions decreased NEDD4-2 ubiquitination in responders. Since NEDD4-2 itself is a substrate of the ubiquitin-proteasome system, which is negatively regulated by phosphorylation [17,49], AMPAR antagonists may increase NEDD4-2 protein level by inhibiting its ubiquitination. Compatible with NEDD4-2 protein level, furthermore, GRIA1 ubiquitination was reduced in epilepsy rats as compared to control animals, which was restored by both AMPAR antagonists in responders. Although it could not be excluded that the higher clearance of AMPAR antagonists by multidrug efflux systems or the lower affinities of AMPAR antagonists on GRIA1 due to allosteric changes of AMPAR [23,24,25] would decrease the efficacies of AMPAR antagonists in non-responders, our findings indicate that maladaptive regulation of intracellular signaling pathways for NEDD4-2-mediated GRIA1 ubiquitination may be one of the important factors in pharmacoresistant seizures to AMPAR antagonists.

The binding of NEDD4-2 to substrates is differently regulated by phosphorylation. In the unphosphorylated state, NEDD4-2 binds to epithelial Na^+^ channels (ENaC), Na_v_1.6, KCNQ2/3 and KCNQ3/5, and facilitates their ubiquitination [12,13,14,15]. In the phosphorylated condition, however, NEDD4-2 preferentially potentiates the degradations of SGK1, GRIA1 and glutamate transporter-1 (GLT-1) [11,17,36,50]. Thus, it is likely that the phosphorylation status may contribute to switching the downstream substrate specificity of NEDD4-2, which is pending further investigation. SGK1 is a serine–threonine kinase that plays an important role in NEDD4-2 phosphorylation [51]. Interestingly, the enhanced SGK1-mediated NEDD4-2 phosphorylation decreases SGK1 protein levels in a dose-dependent manner by increasing SGK1 ubiquitination/degradation in the 26S proteasome. Thus, SGK1 and NEDD4-2 regulate one another in a reciprocal manner [36]. SGK1 protein level is upregulated in the temporal neocortex of patients with pharmacoresistant epilepsy and chronic epilepsy rats [52]. Consistent with this report, the present study shows that SGK1 protein level was increased, while its ubiquitination was reduced, in the epileptic hippocampus as compared to the normal (control) hippocampus. Furthermore, AMPAR antagonists restored SGK1 level to control level by increasing its ubiquitination and NEDD4-2 protein level in responders. Considering the reciprocal SGK1-NEDD4-2 interactions [36], these findings indicate that SGK1 upregulation may be relevant to the NEDD4-2 degradation in the epileptic hippocampus, which would be attenuated by AMPAR antagonists.

SGK1 is activated by phosphorylating S78 and S422 sites through MAPK-ERK1/2 and PI3K-PDK1-AKT pathways, respectively [37,38,39,40]. NEDD4-2 can also be directly phosphorylated by AKT [41]. Consistent with previous studies [4,10,30], the epileptic hippocampus showed upregulated PDK1-AKT phosphorylation and downregulated ERK1/2 phosphorylation, which were reversely regulated by AMPAR antagonists. However, both SGK1 S78 and S422 phosphorylation ratios were lower in epilepsy rats than those in control animals. In addition, AMPAR antagonists enhanced S78, but not S422, phosphorylation level in responders, although they inhibited the PDK1-AKT signaling pathway. Taken together, our findings suggest that reduced ERK1/2 activity (phosphorylation) may lead to dysregulation of SGK1-mediated NEDD4-2 activation, independent of PI3K-PDK1-AKT signaling cascade.

In the present study, PP2A and PP2B expressions in chronic epilepsy rats were lower than those in control animals. Furthermore, their phosphorylation ratios were also reduced in epilepsy animals. Since phosphorylation inhibits protein phosphatase activities [45,46], it is likely that the reduced phosphorylation ratios of PP2A and PP2B may be compensatory responses for maintenance of their activities against downregulation of expressions. Consistent with our previous study [30], furthermore, both AMPAR antagonists elevated PP2B phosphorylation in responders, indicating the decreased PP2B phosphatase activity. Considering that PP2A and PP2B deactivate ERK1/2 kinase activity [42,43], these findings indicate that AMPAR antagonists may enhance ERK1/2 phosphorylation by inhibiting PP2B activity. Indeed, CsA co-treatment improved the anti-epileptic effects of AMPAR antagonists in non-responders, concomitant with the increases in ERK1/2 and SGK1 S78 phosphorylation, NEDD4-2 protein level, NEDD4-2 S448 phosphorylation and ubiquitination of NEDD4-2 and GRIA1. Taken together, these findings indicate that dysregulation of PP2B-ERK1/2-SGK1 signaling pathway may play an important role in the generation of refractory seizures to AMPAR antagonists via impaired NEDD4-2-mediated GRIA1 ubiquitination. 

PP2A also leads to SGK1 inactivation [44]. In the present study, however, neither AMPAR antagonist affected PP2A expression or its phosphorylation. Recently, we have reported that AMPAR antagonists inhibit casein kinase 2 (CK2) that binds to PP2A and increases PP2A activity [53]. Thus, the possibility that AMPAR antagonists may reduce CK2-PP2A-mediated SGK1 dephosphorylation in a phosphorylation-independent manner cannot be excluded.

Under physiological conditions, AMPARs contain the GRIA2 subunit, which are permeable to Na^+^ and K^+^, but not Ca^2+^ [54]. In chronic epilepsy rats, membrane GRIA1/GRIA2 ratio is significantly higher than that in control animals, indicating a preponderance of GRIA2-lacking (Ca^2+^-permeable) AMPAR [4,10,26]. A higher expression of Ca^2+^-permeable AMPAR in the epileptic hippocampus results in a subsequent elevated Ca^2+^ influx followed by PP2B activation [55,56,57]. AMPAR antagonists decrease GRIA1/GRIA2 ratio in responders by reducing GRIA1, but not GRIA2, surface expression [4,10,26]. The present study also demonstrates that AMPAR antagonists increased GRIA1 ubiquitination in responders, which indicates the decreased Ca^2+^-permeable AMPAR level. Furthermore, the hyper-activation of AKT-glycogen synthase kinase 3β (GSK3β)-Ca^2+^/cAMP response element-binding protein (CREB) pathway leads to increased Ca^2+^-permeable AMPAR in non-responders [20,26], suggesting that dysregulation of AKT/GSK3β/CREB-mediated GRIA1 surface expression may also be responsible for the prolonged PP2B activation. Therefore, it is plausible that the increased Ca^2+^-permeable AMPAR expression would be a fundamental reason for the lack of response to the AMPAR antagonists in the non-responders. However, the present data show that the lower efficacies of AMPAR antagonists to the PP2B-ERK1/2-SGK1-NEDD4-2-mediated GRIA1 ubiquitination and the inhibition of spontaneous seizure activities (presumably due to the higher clearance of AMPAR antagonists or the lower affinities of AMPAR antagonists on GRIA1 [23,24,25]) in non-responders were improved by CsA co-treatment. If the upregulated GRIA2-lacking AMPAR expression resulted in refractory seizures to AMPAR antagonists, CsA co-treatment would not inhibit seizure activity in non-responders, since a lack of effects of CsA on neuronal excitability and seizure activity is well known [30,58,59]. Therefore, our findings suggest that the upregulated GRIA2-lacking AMPAR expression in non-responders may not be a primary cause of intractable seizures to AMPAR antagonists (although it may be relevant to ictogenesis), but may be a consequence of the irresponsiveness to AMPAR antagonists. Moreover, PP2B inhibition may enhance the efficacies of AMPAR antagonists in non-responders by recovering the dysregulation of ERK1/2-SGK1-NEDD4-2-mediated GRIA1 ubiquitination and secondarily reducing Ca^2+^-permeable AMPAR expression.

On the other hand, deubiquitination (the removal of ubiquitin moieties) also plays a role in AMPAR-mediated neurotransmission [60,61]. Ubiquitin-specific protease (USP) 46, a deubiquitinating enzyme, inhibits GRIA1 ubiquitination, accompanied by a decreased rate in GRIA1 degradation and an increase in AMPAR synaptic accumulation [60]. With respect to this, it is likely that downregulated GRIA1 deubiquitination may be involved in the intractable seizures to AMPAR antagonists, and CsA co-treatment would also inhibit deubiquitinases of GRIA1 in non-responders via unknown mechanisms. However, retigabine, an AED and a Kv7 channel opener, alleviates the acute stress-induced GRIA1 downregulation by increasing USP2 expression [61]. Therefore, further studies are needed to elucidate the role of GRIA1 deubiqutination in intractable seizures to AMPAR antagonists. 

## 5. Conclusions

The present study reveals that AMPAR antagonists ameliorated spontaneous seizure activity by affecting the PP2B-mediated ERK1/2-SGK1-NEDD4-2 signaling pathway, which is relevant to the enhanced GRIA1 ubiquitination. In addition, the dysregulation of this pathway was one of the causes of refractory seizures to AMPAR antagonists. Therefore, our findings suggest that PP2B-ERK1/2-SGK1-NEDD4-2 pathway may be one of the potential therapeutic targets for the treatment of intractable TLE.

## Figures and Tables

**Table 1:** Primary antibodies used in the present study.

Antigen	Host	Manufacturer (Catalog Number)	Dilution Used
NEDD4-2	Rabbit	Abcam (ab131167): IP Abcam (ab46521): WB	1:100 (IP) 1:1000 (WB)
GRIA1	Mouse	Synaptic systems (#182011)	1:100 (IP) 1:1000 (WB)
p-NEDD4-2 S342	Rabbit	Cell signaling (#12146)	1:1000 (WB)
p-NEDD4-2 S448	Rabbit	Abcam (ab168349)	1:1000 (WB)
SGK1	Rabbit	ST John’s Laboratory (STJ25513)	1:100 (IP) 1:1000 (WB)
p-SGK1 S78	Rabbit	Thermo (PA5-38392)	1:1000 (WB)
p-SGK1 S422	Rabbit	Abcam (ab55281)	1:1000 (WB)
Ubiquitin	Rabbit	Abcam(ab7780)	1:1000 (WB)
ERK1/2	Rabbit	Biorbyt (Orb160960)	1:1000 (WB)
p-ERK1/2	Rabbit	Bioss (bs-3330R)	1:1000 (WB)
PDK1	Rabbit	Cell signaling(#3062)	1:1000 (WB)
p-PDK1 S241	Rabbit	Cell signaling (#3061)	1:1000(WB)
AKT	Rabbit	Cell signaling (#9272)	1:1000 (WB)
pAKT S473	Rabbit	Cell signalling (#4060)	1:1000 (WB)
PP2A	Rabbit	Cell signaling (#2038)	1:5000 (WB)
p-PP2A Y308	Rabbit	Sigma (SAB4503975)	1:1000 (WB)
PP2B	Rabbit	Millipore (07-068-I)	1:1000 (WB)
p-PP2B S197	Rabbit	Badrilla (A010-80)	1:1000 (WB)
β-actin	Mouse	Sigma (#A5316)	1:5000 (WB)

IP, immunoprecipitation; WB, Western blot.

## Supplementary Materials

The following are available online at https://www.mdpi.com/article/10.3390/biomedicines9081069/s1; Figure S1: Representative full-gel images of Western blots in Figure 3; Figure S2: Representative full-gel images of Western blots in Figure 4; Figure S3: Representative full-gel images of Western blots in Figure 5; Figure S4: Representative full-gel images of Western blots in Figure 6; Figure S5: Representative full-gel images of Western blots in Figure 8.
