# Supplementary material for: AMPA Receptor Antagonists Facilitate NEDD4-2-Mediated GRIA1 Ubiquitination by Regulating PP2B-ERK1/2-SGK1 Pathway in Chronic Epilepsy Rats
Source: Biomedicines. 2021 Aug 23;9(8):1069. doi: 10.3390/biomedicines9081069 (PMC8391511; doi:10.3390/biomedicines9081069)
Supplement: Supplementary file 1 [file biomedicines-09-01069-s001.zip › biomedicines-1331126-supplementary.pdf]

Supplementary information

AMPA receptor antagonists facilitate NEDD4-2-mediated GRIA1 ubiquitination by regulating PP2B-ERK1/2-SGK1 pathway in chronic epilepsy rats

Ji-Eun Kim*, Duk-Shin Lee, Hana Park, Tae-Hyun Kim, Tae-Cheon Kang*

Department of Anatomy and Neurobiology and Institute of Epilepsy Research, College of Medicine, Hallym University, Chuncheon 24252, Korea

***** Correspondence: Department of Anatomy and Neurobiology and Institute of Epilepsy Research, College of Medicine, Hallym University, Chuncheon 24252, Korea. E-mail: jieunkim@hallym.ac.kr and tckang@hallym.ac.kr; Tel.:+82-33-248-2522 and +82-33-248-2524; Fax: +82-33-248-2525.

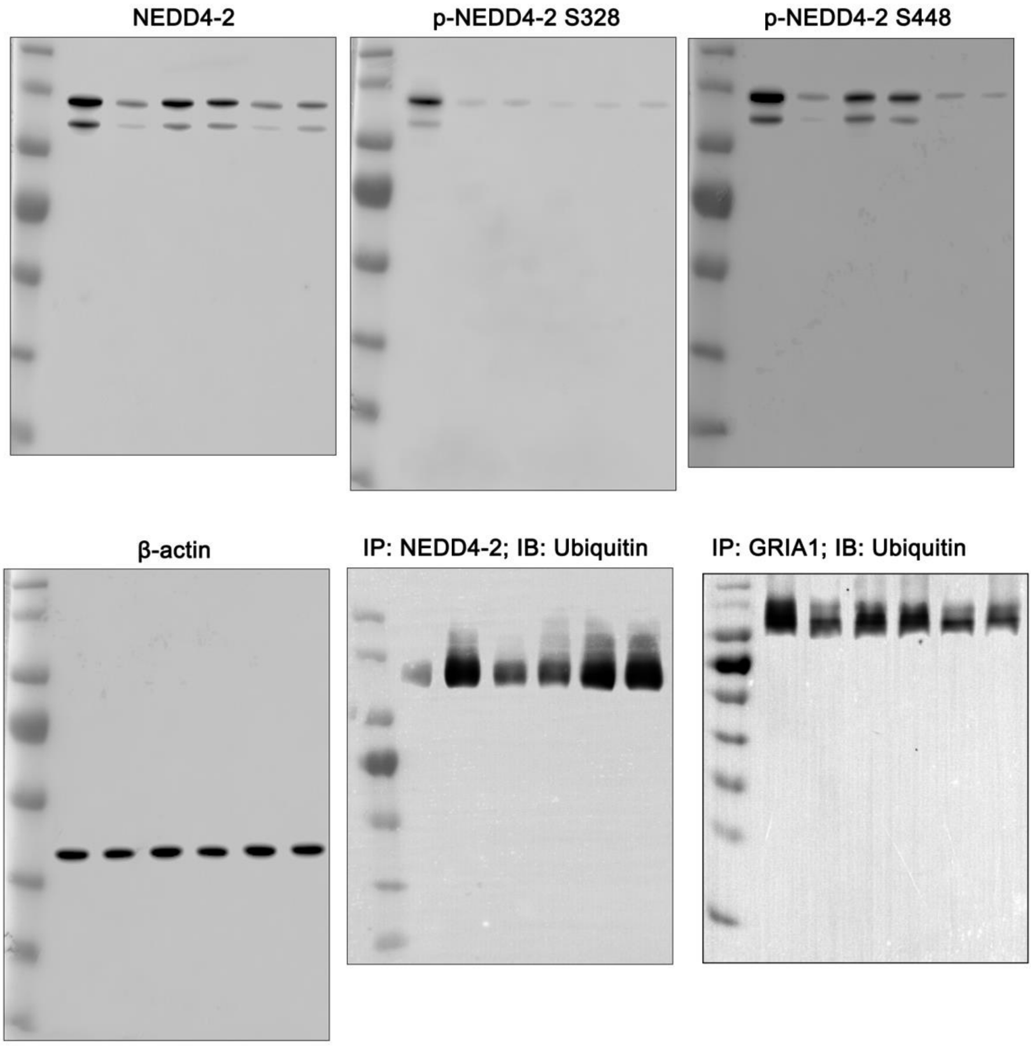

Supplementary Figure S1. Representative full-gel images of Western blots in Figure 3.

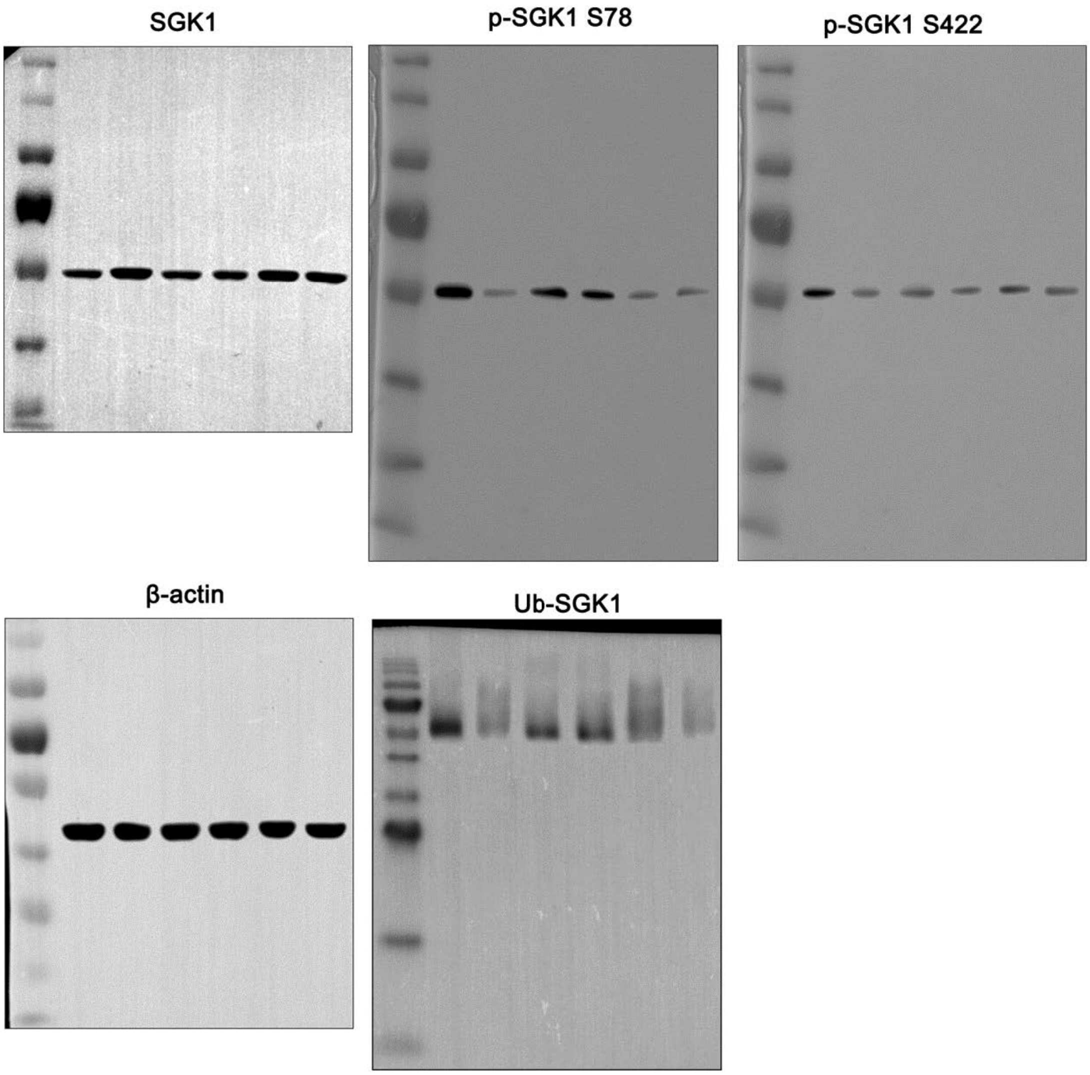

Supplementary Figure S2. Representative full-gel images of Western blots in Figure 4.

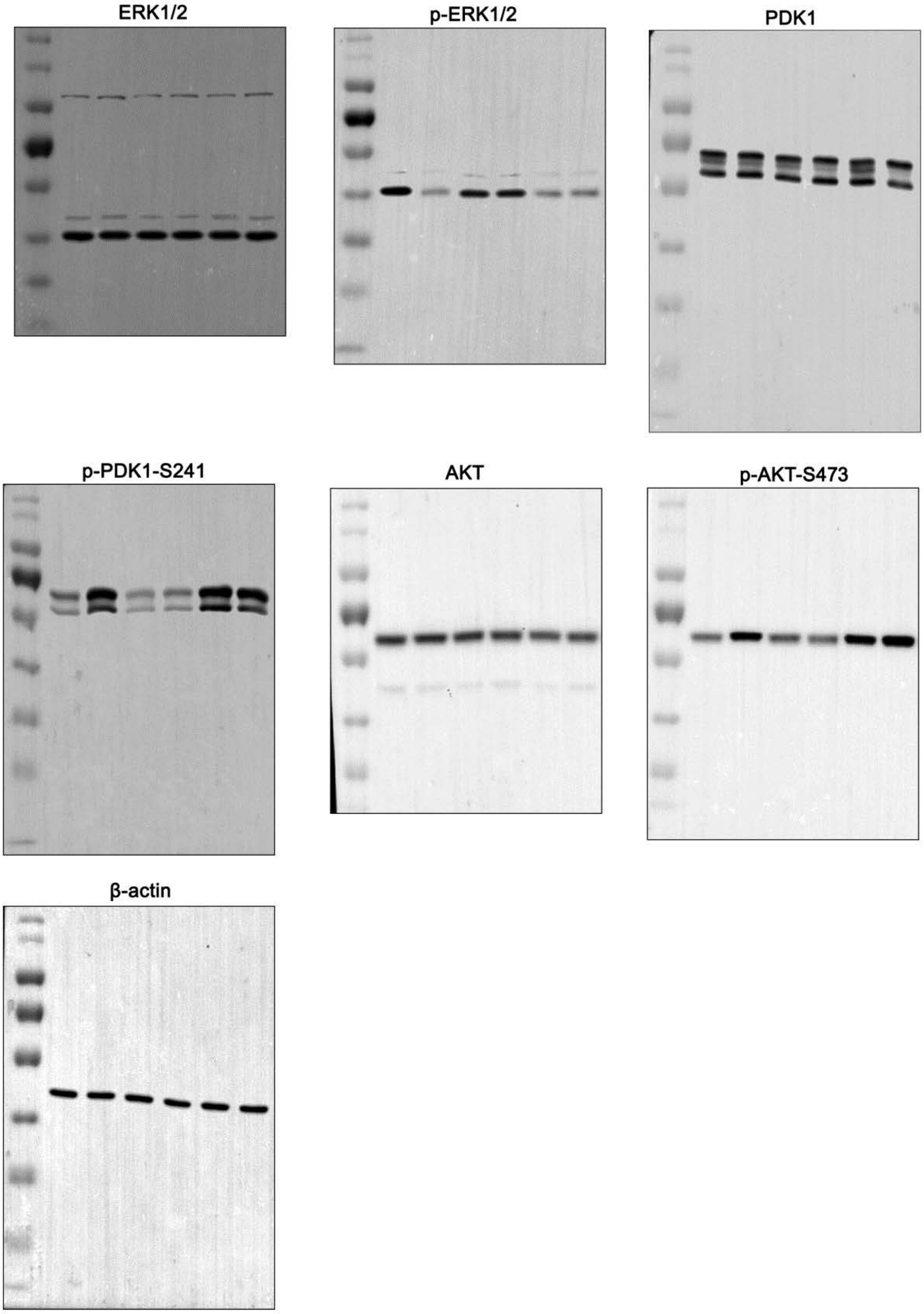

Supplementary Figure S3. Representative full-gel images of Western blots in Figure 5.

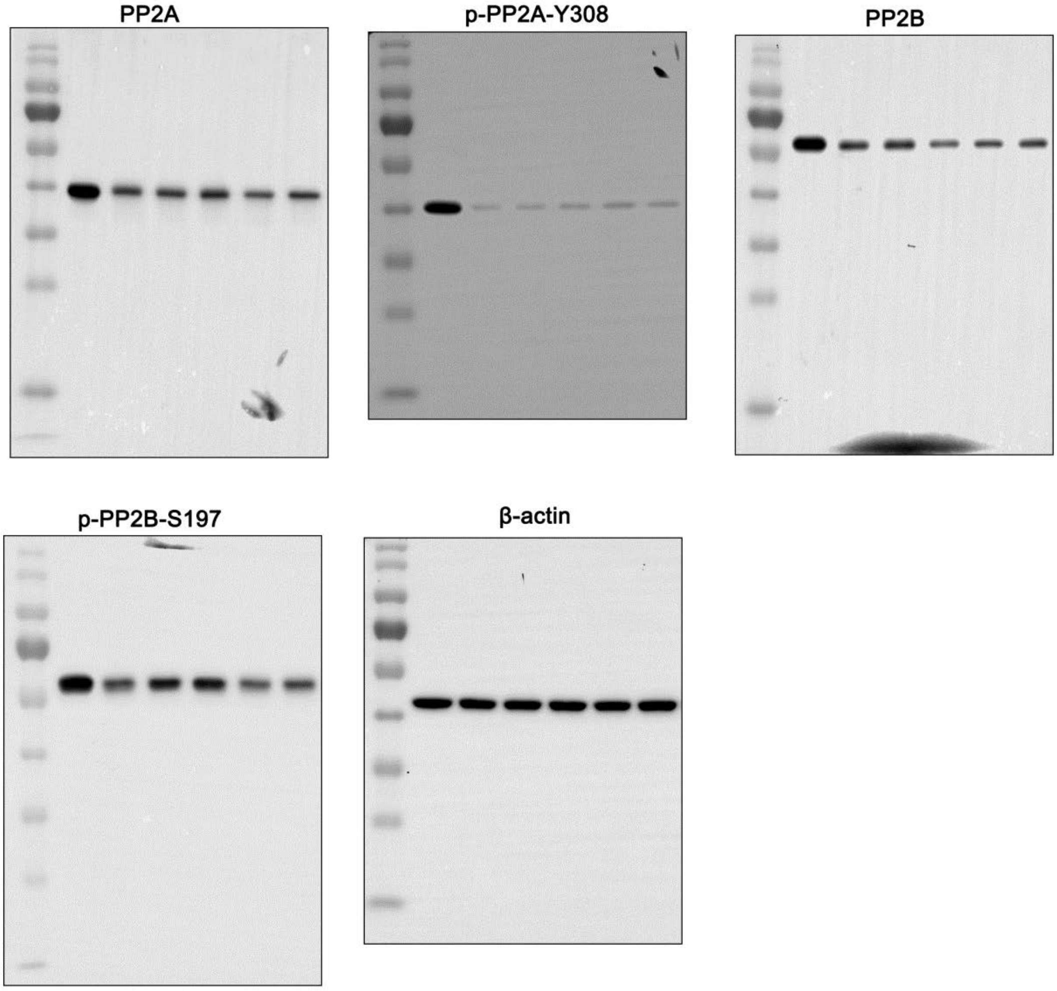

Supplementary Figure S4. Representative full-gel images of Western blots in Figure 6.

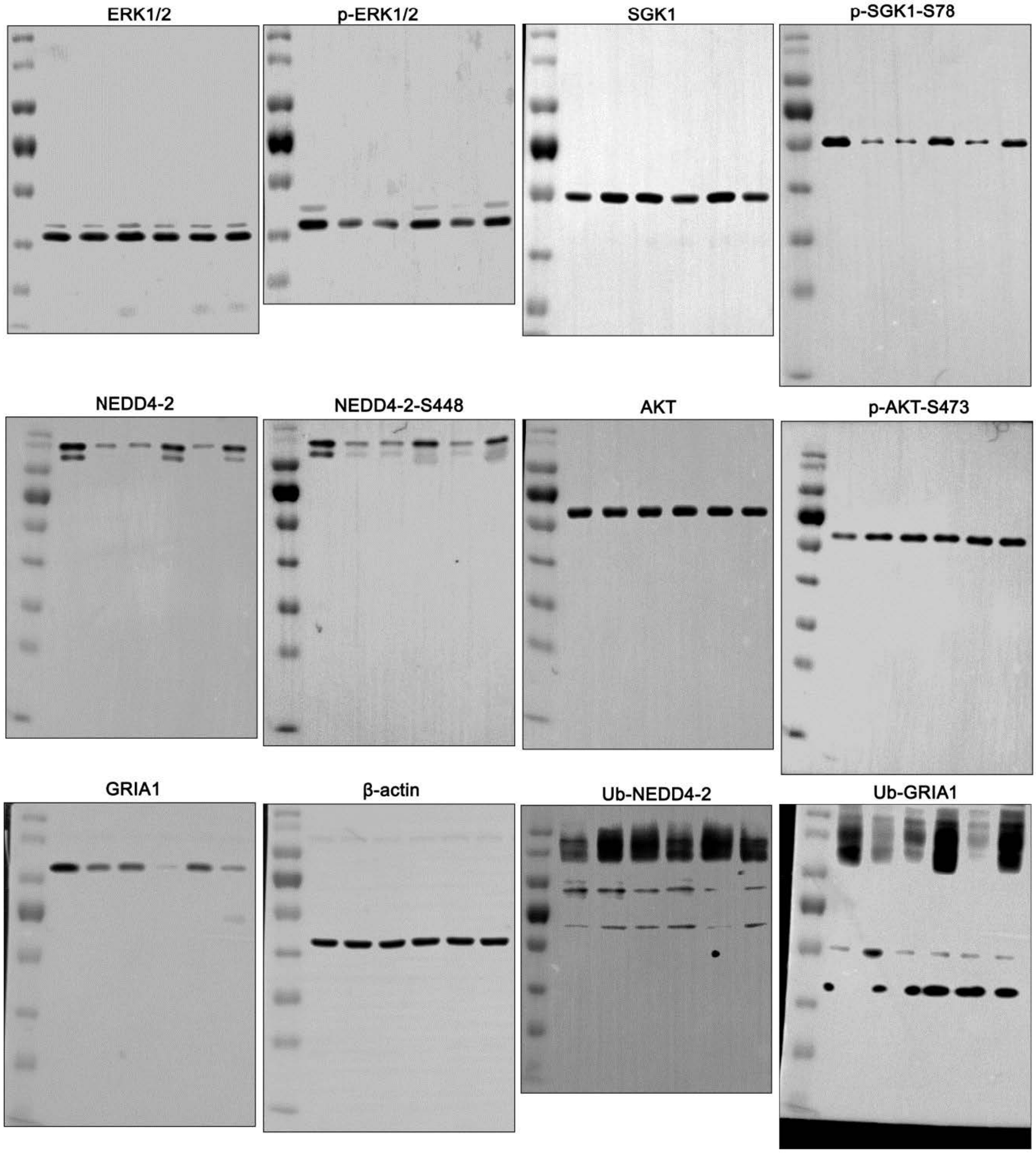

Supplementary Figure S5. Representative full-gel images of Western blots in Figure 8.
